# Genomic pathway analysis reveals that EZH2 and HDAC4 represent mutually exclusive epigenetic pathways across human cancers

**DOI:** 10.1186/1755-8794-6-35

**Published:** 2013-09-30

**Authors:** Adam L Cohen, Stephen R Piccolo, Luis Cheng, Rafaella Soldi, Bing Han, W Evan Johnson, Andrea H Bild

**Affiliations:** 1 Huntsman Cancer Institute, 2000 Circle of Hope, Salt Lake City, UT 84112, USA; 2 Department of Pharmacology and Toxicology, University of Utah, 112 Skaggs Hall, Salt Lake City, UT 84112, USA; 3 Division of Computational Biomedicine, Boston University School of Medicine, Boston, MA, USA

**Keywords:** Epigenetics, Histone acetylation, Histone methylation, mRNA microarray

## Abstract

**Background:**

Alterations in epigenetic marks, including methylation or acetylation, are common in human cancers. For many epigenetic pathways, however, direct measures of activity are unknown, making their role in various cancers difficult to assess. Gene expression signatures facilitate the examination of patterns of epigenetic pathway activation across and within human cancer types allowing better understanding of the relationships between these pathways.

**Methods:**

We used Bayesian regression to generate gene expression signatures from normal epithelial cells before and after epigenetic pathway activation. Signatures were applied to datasets from TCGA, GEO, CaArray, ArrayExpress, and the cancer cell line encyclopedia. For TCGA data, signature results were correlated with copy number variation and DNA methylation changes. GSEA was used to identify biologic pathways related to the signatures.

**Results:**

We developed and validated signatures reflecting downstream effects of *enhancer of zeste homolog 2*(EZH2), *histone deacetylase*(HDAC) 1, HDAC4, *sirtuin 1*(SIRT1), and *DNA methyltransferase 2*(DNMT2). By applying these signatures to data from cancer cell lines and tumors in large public repositories, we identify those cancers that have the highest and lowest activation of each of these pathways. Highest EZH2 activation is seen in neuroblastoma, hepatocellular carcinoma, small cell lung cancer, and melanoma, while highest HDAC activity is seen in pharyngeal cancer, kidney cancer, and pancreatic cancer. Across all datasets studied, activation of both EZH2 and HDAC4 is significantly underrepresented. Using breast cancer and glioblastoma as examples to examine intrinsic subtypes of particular cancers, EZH2 activation was highest in luminal breast cancers and proneural glioblastomas, while HDAC4 activation was highest in basal breast cancer and mesenchymal glioblastoma. EZH2 and HDAC4 activation are associated with particular chromosome abnormalities: EZH2 activation with aberrations in genes from the TGF and phosphatidylinositol pathways and HDAC4 activation with aberrations in inflammatory and chemokine related genes.

**Conclusion:**

Gene expression patterns can reveal the activation level of epigenetic pathways. Epigenetic pathways define biologically relevant subsets of human cancers. EZH2 activation and HDAC4 activation correlate with growth factor signaling and inflammation, respectively, and represent two distinct states for cancer cells. This understanding may allow us to identify targetable drivers in these cancer subsets.

## Background

Epigenetic changes beyond DNA methylation have been recently recognized as important in human cancers [1]. These epigenetic changes include histone modifications such as acetylation and methylation. Histone acetylation is mediated by a balance between histone acetyltransferases (HATs) and the three classes of histone deacetylases (HDACs): Class 1 (HDAC1-3,8), class 2 (HDAC4-7,9-11), and class 3 (Sirt1-7). Histone methylation is mediated by the balance between histone methylases and demethylases. Enhancer of zeste homlog 2 (EZH2), a member of the polycomb repressor complex, is a histone methylase that acts specifically at lysine 27 of histone 3 [2].

Histone acetylation and methylation are altered in multiple cancers, usually with increased histone deacetylation and methylation [3]. Two HDAC inhibitors have been approved for the treatment of T-cell lymphomas, and EZH2 depleting drugs, such as DZNep, have anticancer activity in vitro for multiple tumor types. While drugs targeting these pathways are in development or clinical trials, a detailed map of epigenetic pathway activity in cancer and their relationships to each other remains elusive. Furthermore, the biological phenotypes driven by each distinct epigenetic pathway in cancer have been challenging to discover due to the complex interplay among these enzymes. Measuring their biologic activity in a laboratory setting is also difficult because many of their effects may be modulated through acetylation or methylation of the lysine groups of nonhistone proteins in the cytoplasm, such as p53. The effects of histone acetylation and methylation can vary from location to location in the genome based on other surrounding epigenetic marks. Finally, although target lysines are known for histone methylases such as EZH2, the specific targets of different HDACs are not known.

In this study, we use gene expression patterns to explore the activation of various epigenetic pathways across human cancers. We capture the acute downstream consequences of gene deregulation by isolating RNA directly after a given pathway has been activated and then performing gene expression analysis. We use mRNA to measure the acute changes in gene transcription, which integrates all of the signaling effects of an enzyme. For epigenetic enzymes, these effects can include modification of both histones and other proteins by acetylation, methylation and phosphorylation. Coupling of the signaling pathways to transcriptional responses is a sensitive and accurate reflection of overall pathway activity [4]. We developed gene expression signatures for a prototypical class 1 HDAC (HDAC1), class 2 HDAC (HDAC4), class 3 HDAC (Sirt1), histone methylase (EZH2), and tRNA methylase (DNMT2). We apply these signatures to large public gene expression datasets from multiple cell lines and primary tumors. We demonstrate that some tumor types, such as neuroblastoma, have consistently high EZH2 activation, while pharyngeal cancer and subsets of glioblastoma, non-small cell lung cancer (NSCLC), and breast cancer have high HDAC4 activation. Looking within tumor types, high HDAC4 activation was seen in basal breast cancer and mesenchymal glioblastoma (GBM), while high EZH2 activation was seen in luminal breast cancer and proneural GBM. These analyses led to the novel conclusion that activation of HDAC4 and the histone methylase EZH2 are mutually exclusive and represent two distinct biologic fates in cancer cells, one related to growth factor signaling and the other related to inflammatory signaling.

## Methods

### Epigenetic signature generation

We used human mammary epithelial cell (HMEC) cultures to develop the epigenetic pathway signatures, as these cells have been used previously to generate robust pathway signatures that are accurate across tissue and cancer types [4]. The HMECs were derived from reduction mammoplasties at the University of Utah from patients who provided informed consent under a protocol approved by the University of Utah Institutional Review Board and performed in accordance with principles of the Helsinki Declaration. Recombinant adenoviruses were used to express the protein of interest or Green Florescent Protein (GFP) for controls in HMECs made quiescent by serum starvation. Eighteen hours after infection, cells were collected for both RNA and protein isolation, and expression of HDAC1, HDAC4, SIRT1, DNMT2, and EZH2 were determined by standard Western blotting (Additional file 1: Figure S1). Eighteen hours was chosen based on prior work showing that gene expression changes at this timepoint accurately capture pathway activity [4]. RNA from multiple independent infections was collected for microarray analysis using the Affymetrix Human Genome U133 microarray platform. Microarray data were normalized using the MAS 5.0 algorithm via Affymetrix Expression Console Software Version 1.0 software and then log-transformed and quantile normalized. To standardize expression data for the development of regression models, distance weighted discrimination (DWD) was applied to correct batch effects [5].

Before statistical modeling, gene expression data were filtered to exclude probe sets with signals present at low levels and for probe sets that did not vary significantly across samples. A Bayesian binary regression algorithm was then used to generate multigene signatures that distinguish activated cells from controls [6]. Detailed descriptions of the statistical methods and parameters for individual signatures are given in Additional file 2 Methods. In brief, a multigene signature was developed to represent the activation of a particular pathway based on first identifying the genes that varied in expression between the control cells and the cells with the pathway active. The expression of these genes in any sample was then summarized as a single value or metagene score corresponding to the value from the first principal component as determined by singular value decomposition (SVD). Given a training set of metagene scores from samples representing two biological states (for example, pathway-activated and quiescent control), a binary probit regression model was estimated using Bayesian methods. Applied to metagene scores calculated from gene expression data from a new sample, the model returned a probability for that sample being from either of the two states, which is a measure of how strongly the pathway was activated or repressed in that sample on the basis of the gene expression pattern [6]. When comparing results across datasets, pathway activity predictions from the probit regression were log-transformed and then linearly transformed within each dataset to span from 0 to 1.

### Testing and validation of pathway signature accuracy

To validate pathway signatures, two types of analyses were performed. First, a leave-one-out cross validation (LOOCV) was used to confirm the robustness of each signature to distinguish between the two phenotypic states,GFP versus pathway activation. Model parameters were chosen to optimize the LOOCV and then fixed. Secondly, an *in silico* validation analysis was performed using external and independently generated datasets with known pathway activation status based on biochemical measurements of protein knockdown (SIRT1, HDAC1), inhibitor treatment (HDAC1, DNMT2, EZH2), or activator treatment (HDAC4, SIRT1). A pathway signature’s ability to correctly predict pathway status in these datasets was used to validate the accuracy of the genomic model.

### Tumor datasets

Publically available datasets from Gene Expression Omnibus (GEO) [7] and ArrayExpress [8] were downloaded if they satisfied the following conditions: samples included human primary tumors, the Affymetrix U133 platform was used (to avoid cross-platform signal loss), and either raw CEL files or MAS 5.0 normalized data were available. When CEL files were available, MAS 5.0 normalization was performed. Individual samples for which the ratio of expression for the 3’ and 5’ end of the GAPDH control probes was greater than 3 were considered potentially degraded and removed. The selected datasets are described in Additional file 3: Table S1.

The statistical methods used here to develop gene expression signatures of pathway activity have been previously described [4] and are described in detail in the Additional file 2 Methods. Detailed descriptions of the generation and validation of each pathway signature are available in the Additional file 2 methods. All code and input files are available http://io.genetics.utah.edu/files/bildres/Epigenetics/. All pathway analyses were performed in R version 2.7.2 or MATLAB. Survival analyses were performed using Cox proportional hazards regression with pathway activation as a continuous variable (http://www.statpages.org/prophaz.html).

### Gene set enrichment analyses

GSEA was performed using Gene Set Enrichment Analysis v2 sofware downloaded from the Broad Institute (http://www.broadinstitute.org/gsea) [9,10]. Gene sets from the c2, c4, c5, and c6 collections in MsigDB v3.1 [9] were used.

Breast cancer and glioblastoma copy number data were downloaded via The Cancer Genome Atlas (TCGA) data portal to identify genes with a log2 tumor-normal ratio greater than 0.5 or less than -0.5 in at least 20% of the two subgroups of interest. Commonly altered genes for each cancer were eliminated by filtering out genes with copy number alterations in both subgroups. Gene lists were then analyzed for chromosomal location as well as Gene Ontology (GO) and KEGG pathways using GATHER [11]. Methylation data were preprocessed using Universal Probability Codes and differentially methylated sites were identified using a sliding-window-based paired *t*-test between the two subgroups of interest. Genes with p < 0.1 were kept. The rate of false positives was then estimated by randomly shuffling sample labels 100 times.

## Results and discussion

### Generation of epigenetic pathway signatures

**Figure 1:**
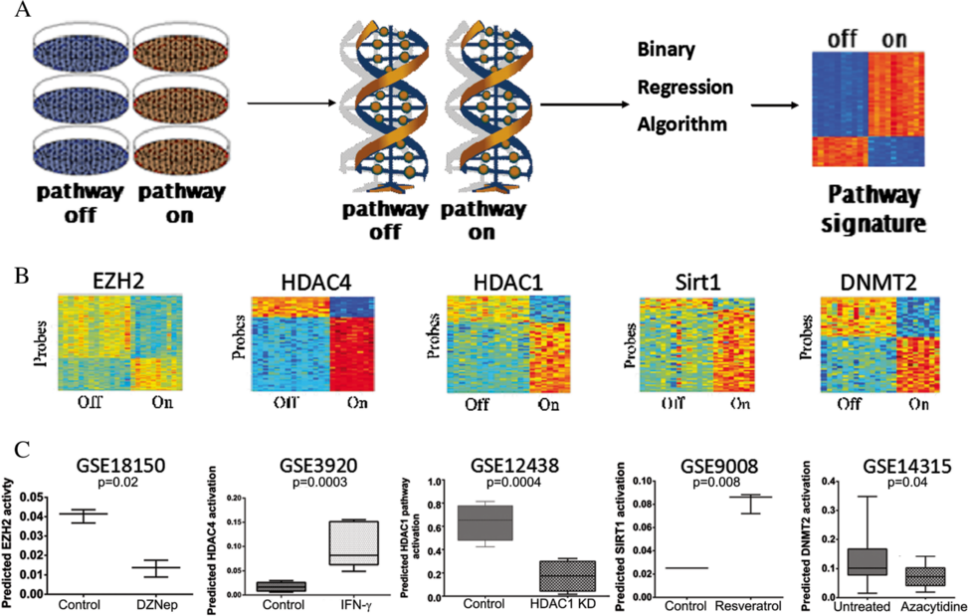
**Genomic signatures of epigenetic pathways. A**. Schematic representation of the process used to generate pathway signatures, first by virally transfecting human mammary epithelial cells and then isolating RNA for microarray analysis followed by the binary regression algorithm to generate the signature. **B**. Heatmaps for the epigenetic pathway signatures. In the heatmaps, each column is a sample, with GFP controls on the left, and each row is a probe. Red indicates increased expression and blue indicates low expression. **C**. External in silico signature validation. Publically available datasets generated by independent groups were used to validate the signatures.

In order to model epigenetic processes in tumors, we used a previously described and validated method for generating genomic pathway signatures (Figure 1A) [4]. Briefly, genes are overexpressed in senescent primary epithelial cells to activate a specific signaling pathway. Following pathway activation, we perform gene expression analysis to capture the acute transcriptional events that are dependent upon that pathway’s activity. Bayesian statistical methods are used to develop pathway-specific gene expression signatures, which are applied to tumor gene expression datasets to estimate each pathway’s activity in each patient tumor sample. The advantages of using genomic profiling to estimate pathway activity in tumor samples over standard biochemical methods include the ability to measure multiple pathways simultaneously in an individual sample and the ability to profile a large number of tumors to uncover novel patterns of pathway deregulation.

In order to investigate epigenetic signaling pathways in cancer, we created a panel of gene expression signatures that model histone methylation (EZH2 signature), histone deacetylation by class 1 (HDAC1 signature), class 2 (HDAC4 signature), and class 3 (SIRT1 signature) histone deacetylases, and RNA methylation (DNMT2 signature). (Figure 1B) Internal validation by leave-one-out cross-validation ensures consistency and robustness of the signatures. External validation was carried out by applying the signatures to publically available datasets obtained from GEO and ArrayExpress (Figure 1C). The EZH2 signature was validated by showing significantly lower predicted EZH2 activity in four different datasets: 1) cells treated with the EZH2-depleting drug DZNep in GSE18150, 2) EZH2 siRNA knockdown from EM-EXP1581, 3) cells from EZH2-null mice in GSE20054, and 4) fibroblasts from EZH2-deficient mice from GSE23659. The last three are shown in Additional file 4: Figure S2. The HDAC1 signature was validated by showing significantly lower predicted HDAC1 activity in cells with HDAC1 siRNA knockdown in GSE12438. The HDAC4 signature was validated by showing significantly increased HDAC4 activity in cells treated with interferon gamma, a known upstream activator of HDAC4, in GSE3920. The SIRT1 signature was validated by showing significantly increased predicted SIRT1 activity in cells treated with resveretrol, a known SIRT1 activator, in GSE9008. The DNMT2 signature was validated by showing it predicted lower DNMT2 activity in cells from GSE14315 treated with azacytidine, a hypomethylating agent. Gene lists for each signature are given in Additional file 5: Table S2. As an additional negative control we tested the relationship between predicted pathway activity and proliferation; none of the signatures correlated with gene proliferation in breast cancer cell lines (Additional file 6: Figure S3).

### Patterns of epigenetic pathway activation across cancer types

**Figure 2:**
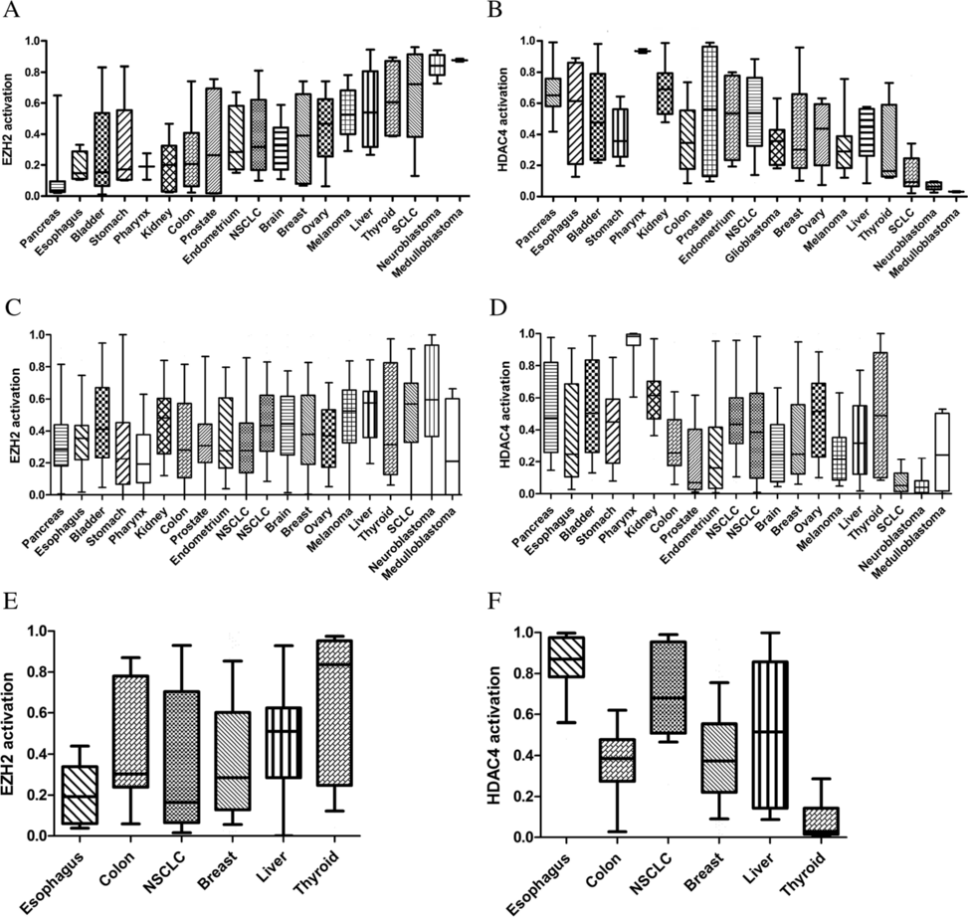
**Epigenetic pathway activation across cancer types.** Epigenetic signatures were projected into the CCLE **(A EZH2 signature, B HDAC4 signature)**, GSK cell lines **(C EZH2 signature, D HDAC4 signature)**, and a dataset of primary tumors from GSE5364 **(E EZH2 signature, F HDAC4 signature)**. Only tumor types represented in both the CCLE and GSK sets are shown. All x-axes are in the same order. Relative activation between different cancer types of each pathway are similar across the datasets.

We first examined the pattern of epigenetic pathway activation across two independent panels of cancer cell lines (Figures 2A- D). The Glaxo-Smith-Kline (GSK) collection profiles 310 cancer cell lines placed on microarrays in one batch (https://array.nci.nih.gov/caarray/project/woost-00041). Over 40 different cancer types are represented, enabling comparisons across cancer type. In all analyses, pathway predictions for replicate samples were averaged. Some cancer types have wide variation in pathway activation, while others have more consistency within cancer type. Strikingly, cancer types with high EZH2 activation consistently also have low HDAC4 activation (Figure 2A and 2B, r_s_ = -0.75, p < 0.000001). This pattern of mutually exclusive and inverse pathway activity was confirmed in a larger dataset of over 900 cell lines from the Cancer Cell Line Encyclopedia (CCLE, shown in Figure 2C and 2D, r_s_ = -0.7, p < 0.0001) [12]. Specifically, in both sets, the more embryonal cancers—neuroblastoma, small cell lung cancer (SCLC), hepatocellular carcinoma (denoted as liver cancer in the figure), and melanoma—had the highest EZH2 activation and lowest HDAC4 activation. Similarly, medulloblastoma had the highest activation of EZH2 and lowest activation of HDAC4 in the GSK dataset but this was not completely replicated in the CCLE. On the other hand, HDAC4 was highest in pharyngeal, kidney, and pancreatic cancers. HDAC1 and SIRT1 also had high consistently activation in pharyngeal,kidney, and liver cancers and low activation in SCLC and neuroblastoma. DNMT2 had higher activation in SCLC, neuroblastoma, and medulloblastoma compared to all other cancers, which were at a similar low level.(Additional file 7: Figure S4).

Many of our cell line results are consistent with published research. For example, neuroblastoma has been shown to have high EZH2 activity and to rely on this activity for survival [13,14]. In addition, upregulation of HDAC4 in neuroblastoma cells changes their proliferation rate, suggesting it is not otherwise active in neuroblastoma [15]. Similarly, EZH2 has recently been shown to be upregulated and active in SCLC [16,17]. Indeed, in a large Japanese series, 67% of SCLC had tumor-to-normal expression ratios for EZH2 of greater than 5, compared with 10% of NSCLC and 6% of esophageal carcinomas [18]. Activation of HDAC4 in hypoxic response of kidney cancer has been described as has high HDAC4 gene expression [19-21].

To investigate pathway activity in actual patient tumors, we then projected the signatures onto a dataset of primary tumor and normal samples (GSE5364, Figure 2E and 2F) [22]. The relative activation of the epigenetic pathways in the thyroid, breast, non-small cell lung, liver, colon, and esophagus cancers mirrored what we saw in the cell lines, confirming the relevance of the patterns seen in the cell lines. Note that the apparent discrepancy between the thyroid cell lines in the CCLE and the other two sets is likely due to the inclusion of anaplastic thyroid cancer cell lines in the CCLE in addition to differentiated thyroid cancer. Consistent with our cell line results and prior studies, hepatocellular carcinoma (HCC) showed high activation of EZH2 and HDAC1 [23-28]. Low DNMT2 expression in HCC has also been previously reported [29]. We describe less activation of HDAC4 in HCC than other cancers. Our results are also consistent with literature showing that most esophageal cancer has low EZH2 levels [30].

Although most prior research has focused on expression levels of individual genes, multi-gene expression signatures may be more accurate than interrogating single-gene mRNA or protein levels. Activation of many signaling pathways, including the epigenetic pathways investigated here, does not always correlate with expression, as pathway activity levels can be determined by many factors, including RNA expression, protein ubiquitination, and expression levels of other proteins in the complexes. Even proposed end readouts of epigenetic pathways, such as H3K27 trimethylation for EZH2, may miss effects of these proteins on non-histone proteins or through other mechanisms [31]. Therefore, gene expression signatures of pathway activation have the potential to give more comprehensive estimates of how active the epigenetic enzymes are than simple expression levels or histone changes.

### Patterns of epigenetic pathway activation within cancer subtypes

**Figure 3:**
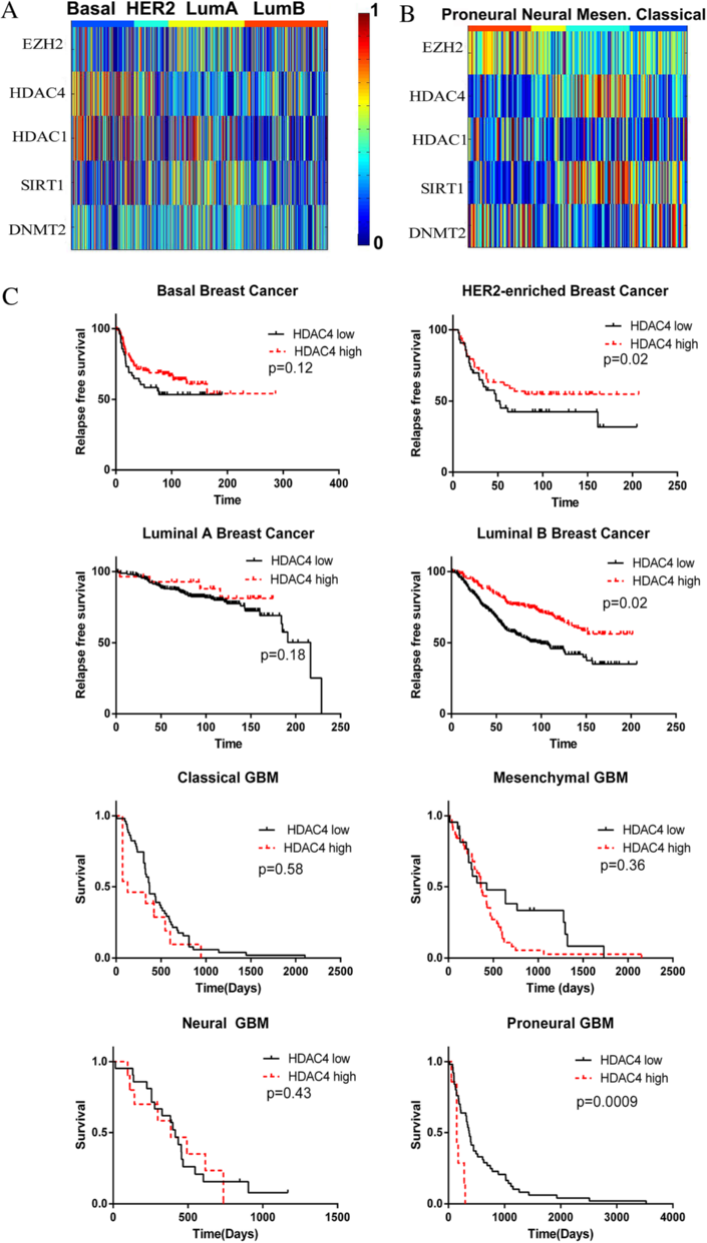
**Epigenetic pathway activation in individual breast and brain cancers.** The same color scale applies to both heatmaps. In each heatmap, each column gives the pathway activation for all 5 epigenetic pathways for an individual tumor. **A**. The epigenetic pathway signatures were projected into a metadataset of breast cancer primary tumors. **B**. The epigenetic pathways were projected into gene expression microarray data from the TCGA glioblastoma project. **C**. Kaplan-Meier survival curves showing improved relapse free survivalwith high HDAC4 activation specific subtypes of breast cancer from the metadataset of breast cancers and decreased survival with high HDAC4 activation in proneural GBM from the TCGA.

Because of the variability in epigenetic pathway activation within certain cancers, we examined the relationship between epigenetic pathway activation and known subtypes of two common cancers with well-defined subtypes: breast cancer and glioblastoma. In order to map epigenetic pathway activity within specific cancer subtypes, we used The Cancer Genome Atlas (TCGA) and other public tumor datasets. Breast cancer subtypes (basal, luminal A, luminal B, and HER2-enriched) have been well described [32,33]. Glioblastoma subtypes (mesenchymal, neural, proneural, and classical) were described in the initial TCGA reports [34]. We first projected the epigenetic pathway signatures into a metadataset of 1492 primary breast cancer samples from 12 different datasets that we had integrated previously (Figure 3A) [35]. Duplicate samples, degraded samples, as well as samples assigned to the normal-like subtype were removed. Subtypes were compared using ANOVA. The basal subtype was characterized by high overall HDAC4 and HDAC1 activity (p ≤ 0.0001 for both). Indeed, 61% of tumors with high HDAC4 and HDAC1 activation were basal. The luminal A subtype was characterized by high EZH2, SIRT1, and DNMT2 activity (p < 0.0001). Overall, 81% of tumors with high EZH2 and low HDAC4 and 83% of tumors with high EZH2 and high SIRT1 activity were luminal. These results are consistent with cell line findings from the CCLE, in which basal breast cancer cell lines had significantly higher HDAC4 activation than luminal cell lines (p = 0.0004) and luminal breast cancer cells had significantly higher EZH2 activation than basal cell lines (p = 0.04).

Although initially our results may seem to contradict other reports that EZH2 is overexpressed in basal breast cancers compared to luminal cancers, there are areas of agreement [36,37]. EZH2 gene expression and pathway activity need not correlate. Indeed, our datasets also had highest EZH2 gene expression in basal breast cancers, despite having highest EZH2 activity in luminal cancers. Moreover, even in reports with high EZH2 expression in basal breast cancers, the activity of EZH2, as measured by the DNA methylation of EZH2 target genes, which is another proposed marker of EZH2 activity because histone methylation leads to DNA methylation, is lowest in basal breast cancers and highest in luminal cancers [36,38,39]. Indeed, EZH2 may be elevated in basal breast cancer through negative feedback because its downstream pathway is inactive. Moreover, others have found that EZH2 directly interacts with the estrogen receptor to assist in activating estrogen-responsive genes [40]. Finally, EZH2 may have context-dependent functions so that it affects different genes, depending on the environment, such as the estrogen-receptor status of a cancer [41]. Therefore, the genes affected by EZH2 modulation may differ in luminal and basal cancers.

Similarly, epigenetic pathway activation varied among GBM subtypes (Figure 3B). Again, ANOVA was used to compare subtypes. EZH2 and HDAC1 pathway activation were highest in the Proneural subtype, while HDAC4 and SIRT1 were highest in the Mesenchymal subtype (p = 1.63 × 10^-5^, 7.6 × 10^-3^, 7.97 × 10^-19^, and 8.04 × 10^-22^, respectively). DNMT2 activation was relatively lower in the Mesenchymal and Neural subtypes compared to the others (p = 2.8 × 10^-7^). Of those GBMs with high EZH2 and high HDAC1 activation, 58% are Proneural, while 73% of GBM with high HDAC4 and SIRT1 activation are Mesenchymal. Although these pathways have not been assessed directly within GBM subtypes before, our results are consistent with the finding that EZH2 expression is highest in secondary GBM, which tend to be Proneural, rather than primary GBM [42].

To assess the potential clinical significance of epigenetic pathway activation, we assessed whether EZH2 activation or HDAC4 activation predicted prognosis in our metadataset of breast cancer or TCGA data of GBM. EZH2 activation was prognostic in neither cancer. HDAC4 activation was not prognostic in breast cancer overall, but higher HDAC4 activation predicted better prognosis when looking within the HER2-enriched (HR 0.29, 95% CI 0.1-0.85) and luminal B (HR 0.43, 95% CI 0.21-0.88) subtypes (Figure 3C). On the contrary, higher HDAC4 activation was a poor prognostic indicator in GBM (HR 1.85, 95% CI 1.09-3.15). Interestingly, this effect seen most strongly within proneural subtype GBM (HR 7.92, 95% CI 2.2-26.9).

### General relationship between epigenetic pathways

**Figure 4:**
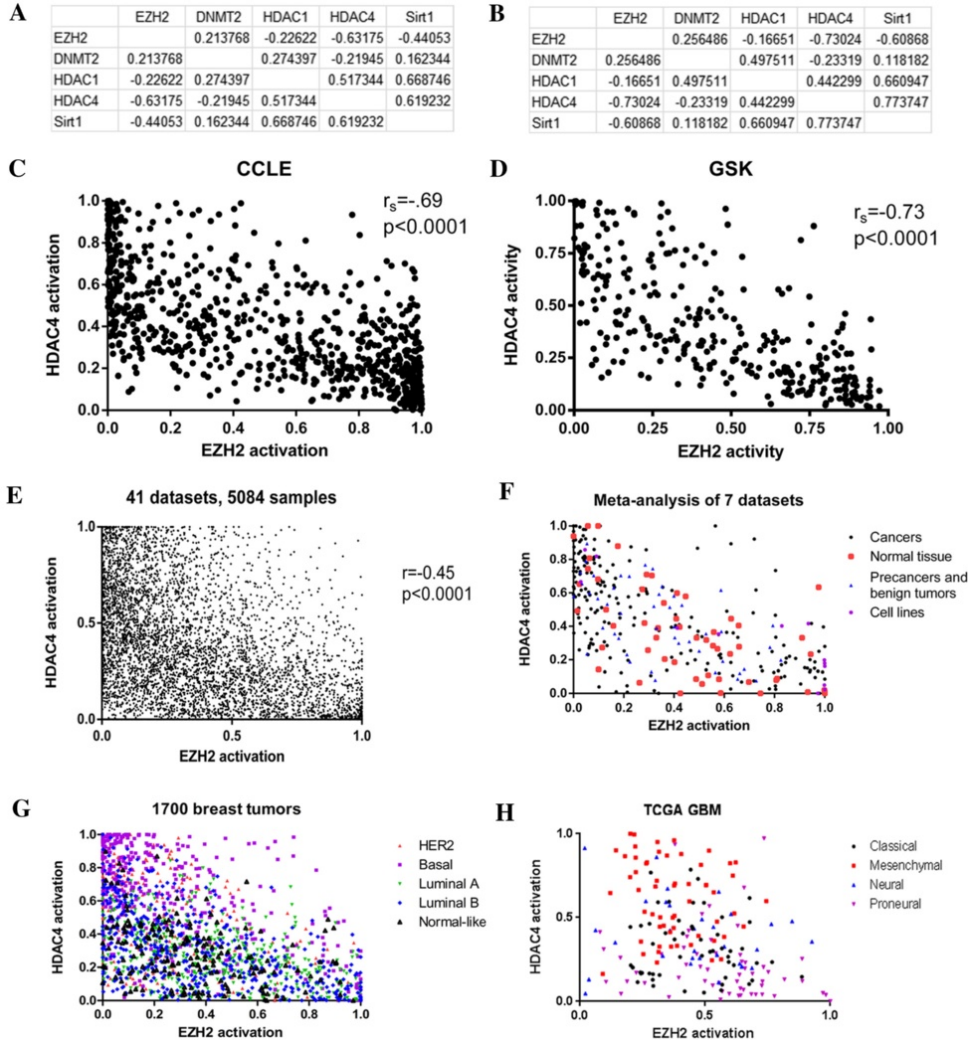
**EZH2 and HDAC4 activation are mutually exclusive. A**. Table of Spearman correlation coefficients for all 5 epigenetic pathways in the CCLE dataset. All coefficients have p < 0.000004. **B**. Table of Spearman correlation coefficients for all 5 epigenetic pathways in the GSK dataset. All coefficients have p < 0.04. **C**. Graph of EZH2 and HDAC4 activation in the 300 CCLE samples. **D**. Graph of EZH2 and HDAC4 activation in the GSK cell line samples. **E**. Graph of EZH2 and HDAC4 activation in over 5000 primary cancers from a wide variety of cancers. There is a significant lack of cancers in the upper right quadrant, showing the rarity of coactivation of EZH2 and HDAC4. **F**. Graph of EZH2 and HDAC4 activation in datasets including precancers, normal tissues, benign tumors, and cell lines, showing that the inverse relationship of EZH2 and HDAC4 is not cancer specific. **G**. Patterns of activation of EZH2 and HDAC4 mirror intrinsic subtypes of breast cancer. **H**. Patterns of activation of EZH2 and HDAC4 mirror subtypes of glioblastoma.

Not surprisingly, there were significant positive correlations between the HDAC1, SIRT1, and HDAC4 pathways (Figure 4A).

These correlations reproduce in the independent GSK dataset, where, again, all p-values are highly significant (Figure 4B). However, surprisingly, as consistent across all data sets was a strong negative correlation between EZH2 and HDAC4. A negative correlation was also seem between EZH2 and SIRT1 in the cell line datasets, but it was not as robustly and consistently seen in human tumor datasets as the EZH2/HDAC4 relationship was. Correlations for individual tumor types are given in Additional file 8: Table S3.

There is a negative correlation between EZH2 activation and HDAC4 activation in both the CCLE and GSK datasets (Figures 4C and 4D). However, the relationship between EZH2 activation and HDAC4 activation is not linear. Rather, although deactivation of both is common, EZH2 activation and HDAC4 activation appear to be mutually exclusive. Figure 4E shows EZH2 and HDAC4 activation in a meta-analysis of 35 publicly available datasets from GEO (listed in Additional file), including over 5000 primary human tumor samples. Only about 3% have activation of both EZH2 and HDAC4, despite an expected rate of 9.5% (p = 1 × 10^-88^). (30% of tumors had activation of HDAC4 only, while 25% had activation of EZH2, and 42% had activation of neither). This exclusion is consistent across cancers of all types, locations, and stages. This relationship is not simply a mathematical artifact of the formulas for the two signatures because it is not seen when the signatures are applied to non-biologically meaningful samples, such as microarrays run on degraded RNA (Additional file 9: Figure S5). Together, these data suggest a strong and consistent inverse relationship between EZH2 and HDAC4 pathways that has previously remain undiscovered.

### Epigenetic pathway exclusivity in cancer and normal tissue

To investigate whether the mutually exclusive relationship between EZH2 and HDAC4 was seen only in cancers, we applied these signatures to 7 datasets that contained a mixture of primary human cancers, cell lines, primary human pre-cancers, and normal tissues that were not adjacent to cancers (Figure 4F). All datasets show a mutually exclusive relationship. Activation of both EZH2 and HDAC4 was rare in cancers, pre-cancers, and in normal tissues.

As discussed above, activation of epigenetic pathways often correlated with cancer subtypes. The mutual exclusion of HDAC4 and EZH2 gives us another way of understanding the relationship between cancer subtypes. Figure 4G shows the distribution of EZH2 and HDAC4 activation across a meta-analysis of 1700 breast tumors (datasets listed in Additional file 3: Table S1). Tumors with high HDAC4 activation and low EZH2 activation tend to be basal, while tumors with low HDAC4 activation and high EZH2 activation tend to be luminal. Figure 4H shows, using the same data as Figure 3B,the distribution of EZH2 and HDAC4 activation across the TCGA GBM samples, demonstrating that Mesenchymal GBM tend to have high HDAC4 activation while proneural GBM tend to have high EZH2 activation.

### Biological phenotypes of EZH2/HDAC4 tumors

To determine the biologic basis for the mutual exclusivity of EZH2 activation and HDAC4 activation, we explored the effect of EZH2 activation and HDAC4 activation in a number of ways. As shown below, the two pathways seemed to represent distinct biologic states, where HDAC4 is related to inflammatory or chemokine signaling and EZH2 relates to signaling from downstream effectors of receptor tyrosine kinases.

**Figure 5:**
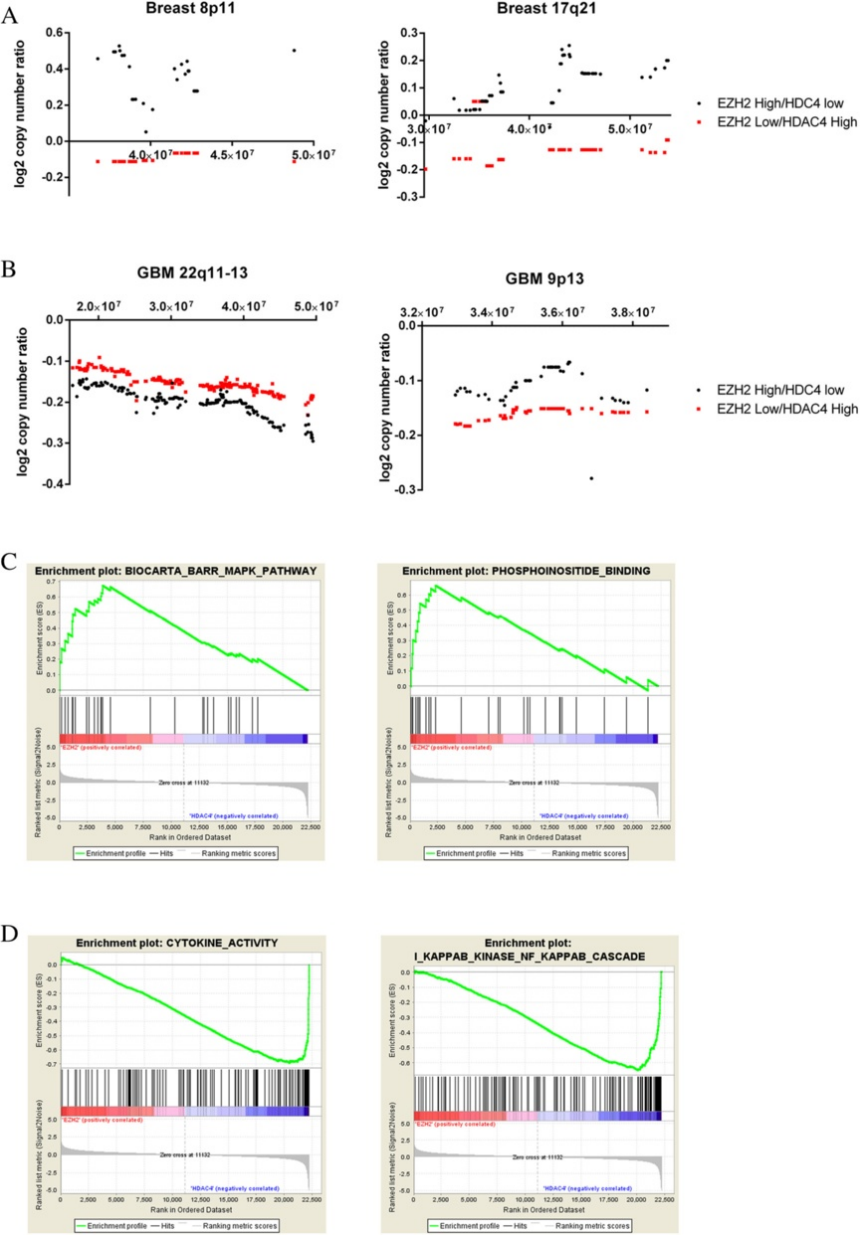
**Biologic correlates of the EZH2 and HDAC4 pathways. A**. Copy number variations in the TCGA breast cancer samples. Each point on the x-axis is a gene. The y-axis gives the average of the log2 ratio of tumor to normal copy number. **B**. Copy number variations in the TCGA glioblastoma cancer samples. Each point on the x-axis is a gene. The y-axis gives the average of the log2 ratio of tumor to normal copy number. **C**. GSEA curves for EZH2 enriched pathways from the PI3K and MAP kinase pathways. **D**. GSEA curves for HDAC4 enriched pathways involved in cytokine and chemokine signaling.

We interrogated the TCGA glioblastoma and breast cancer datasets to investigate pathways enriched in EZH2 or HDAC4 positive tumors. For this analysis, we used the copy number alteration data to link unique genetic variants with epigenetic pathway status. First, we identified two groups of tumors: those with high EZH2 activity and low HDAC4 activity and those with low EZH2 activity and high HDAC4 activity, using a cutoff of 0.5 for GBM and 0.2 for breast cancers. For breast tumors in TCGA, EZH2 low/HDAC4 high tumors are more likely to have copy-number gains in 11q13 and losses in 8p11 and 17q21 and are less likely to have gains in 8p11, 20q11-13, and gains in 17q21 (all Bayes Factors >30). Representative loci are shown in Figure 5A, and the others are shown in Additional file 10: Figure S6. For GBM in TCGA, EZH2 low/HDAC4 high tumors are more likely to have losses of 22q11-13 and gains of 8p11 and17q21 and are less likely to have gains of 5q31(all Bayes Factors > 30). Representative loci are shown in Figure 5B, and the others are shown in Additional file 10: Figure S6. Genes with copy number variation in EZH2 low/HDAC4 high GBM tumors were enriched for genes in the KEGG toll-like receptor pathway and the cytokine-cytokine signaling pathway (Bayes Factors 16-18). These results suggest that the opposing EZH2/HDAC4 pathway activity represents two distinct tumor phenotypes.

In addition to leveraging copy number data, we applied GSEA to the gene-expression data used to generate the EZH2 and HDAC4 signatures to identify pathways associated with either EZH2 activation or HDAC4 activation in the signature samples. EZH2 activation was associated with TGF-beta signaling, phosphatidylinositol binding, and negative regulation of MAPK (Figure 5C). HDAC4 activation was associated with pathways involved in cytokine signaling, inflammation, and infection response (Figure 5D). Similar results were found using GATHER (http://gather.genome.duke.edu) to assess GO and KEGG pathways. Thus, the GSEA results matched the copy-number results, indicating that HDAC4 activation and EZH2 inactivation are associated with increased activation of cytokine and immune-related pathways. These connections between HDAC4 activation and inflammatory cytokines match the cancer subtype results. For example, basal breast cancers, which we found to have high HDAC4 activation, are known to have higher levels of tumor-infiltrating macrophages and higher chemokine receptor expression than luminal cancers [43,44]. Mesenchymal glioblastoma, which we found have higher HDAC4 activation, also have greater infiltration by immune cells than proneural glioblastomas [45]. Alternatively, luminal breast cancers, which have high EZH2 activation, are associated with higher serum TGF levels [46].

Lastly, we used DNA methylation data to investigate further the differences between EZH2 high/HDAC4 low and EZH2 low/HDAC4 high tumors. We identified genes that are differentially methylated between the two groups in the TCGA GBM and breast datasets. With a false discovery rate less than 5%, gene ontology analysis showed that genes with decreased methylation in EZH2 low/HDAC4 high GBM were enriched for T-cell activation (Bayes Factor 5.5). In breast cancer, EZH2 high/HDAC4 low had increased methylation of TNFRSF10D, a stimulator of inflammatory pathways including NF-κB. Thus, the methylation data also show that expression of genes in inflammatory signaling pathways is higher in tumors with high HDAC4 activation than in tumors with high EZH2 activation.

## Conclusions

Using genome-wide gene expression signatures, we have mapped patterns of epigenetic pathway activation in large panels of tumors, enabling discrimination of patterns across and within cancer phenotypes. Looking broadly across all cancers, our results highlight that EZH2 is active in more primitive cancers of childhood, and HDAC4 is active in more mature adenocarcinomas and squamous cell carcinomas. Our analysis indicates two distinct and mutually exclusive types of cancers, one associated with a gene expression pattern of EZH2 activation and tyrosine kinase signaling and the other with HDAC4 activation with increased cytokine signaling and immune cell infiltration. Looking within cancers, epigenetic pathways highlight differences between subtypes of a cancer and similarities between subtypes of different cancers. In particular, EZH2 activation is seen in luminal breast cancers and proneural GBM, while HDAC4 activation is seen in basal breast cancers and mesenchymal GBM. These results raise the possibility for a histology-independent categorization of cancers using epigenetic pathways. Further studies are needed to elucidate the mechanisms for the mutual exclusiveness of EZH2 and HDAC4 and to determine therapeutic targets for the distinct epigenetic-specific cancer phenotypes.

## Abbreviations

HAT: Histone acetyltransferase; HDAC: Histone deacetylase; EZH2: Enhancer of zeste homolog 2; NSCLC: Non-small cell lung cancer; GBM: Glioblastoma; HMEC: Human mammary epithelial cell; GFP: Green fluorescent protein; SVD: Singular value decomposition; LOOCV: Leave-one-out cross-validation; GEO: Gene expression omnibus; GSEA: Gene set enrichment analysis; TCGA: The cancer genome atlas; GO: Gene ontology; GSK: Glaxo-Smith-Kline; CCLE: Cancer cell line encyclopedia; SCLC: Small cell lung cancer.

## Competing interests

The author’s declare that they have no competing interests.

## Author’s contributions

AC performed all bioinformatic analyses, obtained and analyzed datasets, and drafted the manuscript. SP provided software, assisted with analysis, and helped with the manuscript. LC performed western blots. RS performed cell culture, prepared viruses, and confirmed virus transfection. BH performed analysis of methylation data from TCGA. WEJ supervised the methylation analysis, performed some statistical analyses, and helped with the manuscript. AHB conceived of the study, prepared the cells and viruses for the signature, assisted with data interpretation, and helped draft the manuscript. All authors read and approved the final manuscript.

## Pre-publication history

The pre-publication history for this paper can be accessed here:

http://www.biomedcentral.com/1755-8794/6/35/prepub

## Supplementary Material

Additional file 1: Figure S6 Western blot of HMECs infected with viruses expressing epigenetic pathway proteins. Click here for file

Additional file 2: Figure S1 Supplementary methods with detailed instructions for running pathway predictions. Click here for file

Additional file 3: Table 1 Description of all publically available datasets used. Click here for file

Additional file 4: Table S1 Additional external in silico validation graphs for the EZH2 signature using publicallyavailable data. Click here for file

Additional file 5: Figure S2 Gene lists for the five epigenetic pathway signatures. Click here for file

Additional file 6: Table S2 Graph showing the lack of correlation between each of the 5 epigenetic pathway signatres and proliferation, as measured by doubling time, in a panel of breast cancer cell lines. Click here for file

Additional file 7: Figure S3 Epigenetic pathway predictions for HDAC1, DNMT2, and SIRT1 in (A) GSK and (B) CCLE cell line collections. Click here for file

Additional file 8: Figure S4 Table of correlation coefficients for the 5 epigenetic pathway signatures within individual cancer types. Click here for file

Additional file 9: Table S3 Graph of EZH2 and HDAC4 activation in samples obtained from autopsies on the brains of people with Parkinson’s, showing a frequency of coactivation not seen in any dataset of samples from living people. Click here for file

Additional file 10: Figure S5 Copy number variations in the TCGA (A) breast cancer and (B) glioblastoma samples. Each point on the x-axis is a gene. The y-axis gives the average of the log2 ratio of tumor to normal copy number. Click here for file
