# Supplementary figures and images for: Genomic pathway analysis reveals that EZH2 and HDAC4 represent mutually exclusive epigenetic pathways across human cancers
Source: BMC Med Genomics. 2013 Sep 30;6:35. doi: 10.1186/1755-8794-6-35 (PMC3850967; doi:10.1186/1755-8794-6-35)

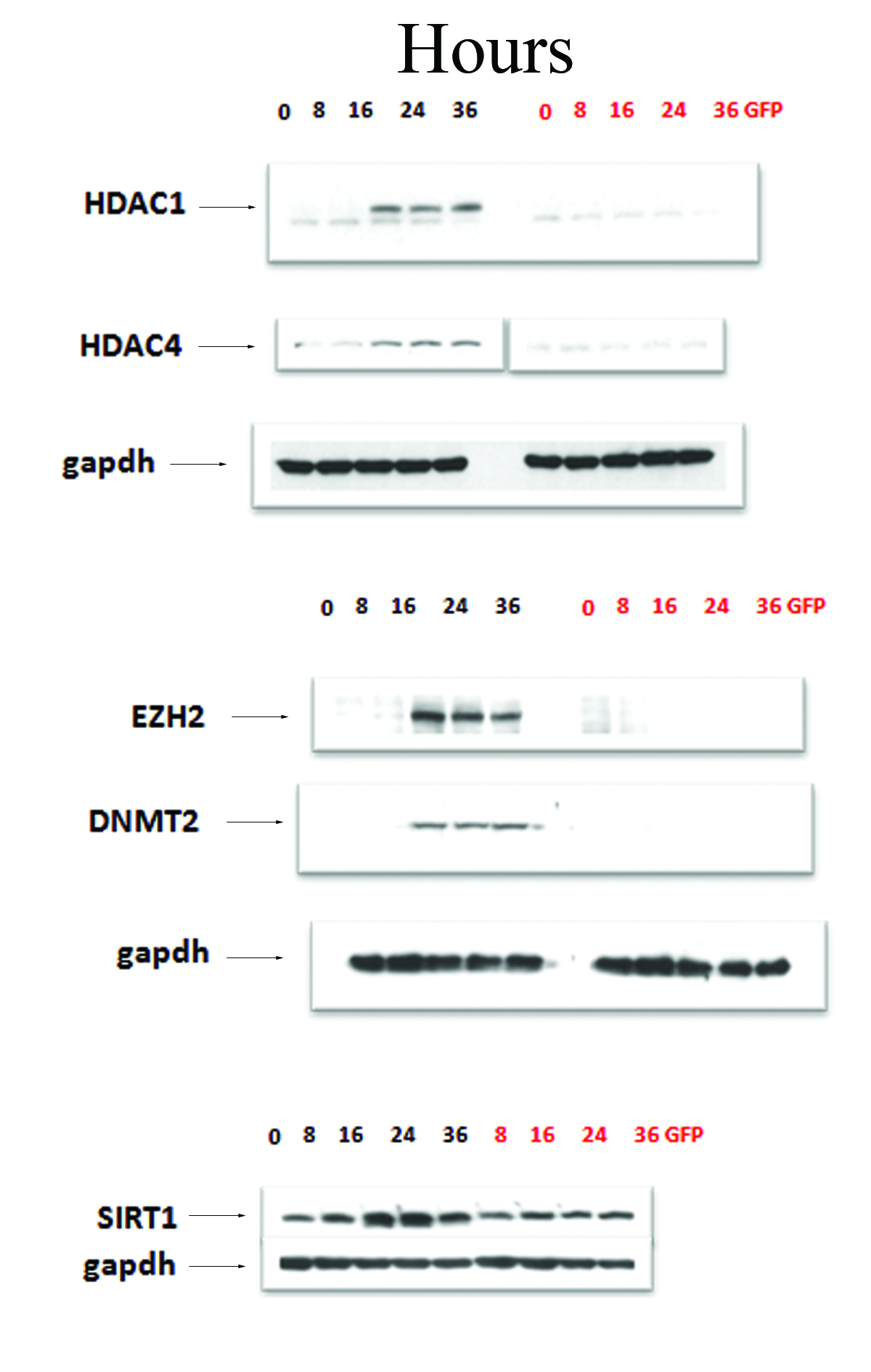

Supplement: Additional file 1: Figure S6 — Western blot of HMECs infected with viruses expressing epigenetic pathway proteins. [file 1755-8794-6-35-S1.tiff]

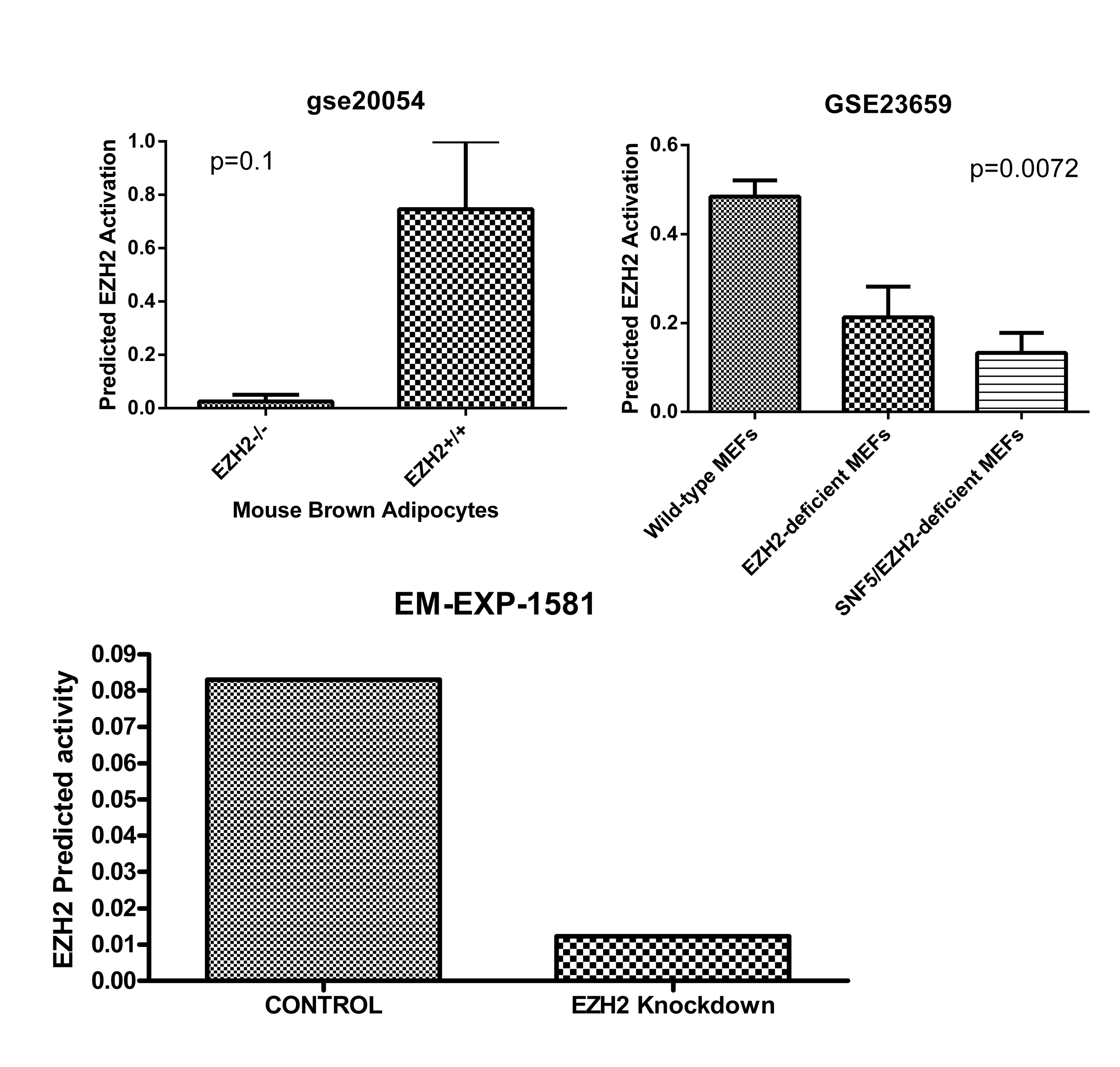

Supplement: Additional file 4: Table S1 — Additional external in silico validation graphs for the EZH2 signature using publicallyavailable data. [file 1755-8794-6-35-S4.jpeg]

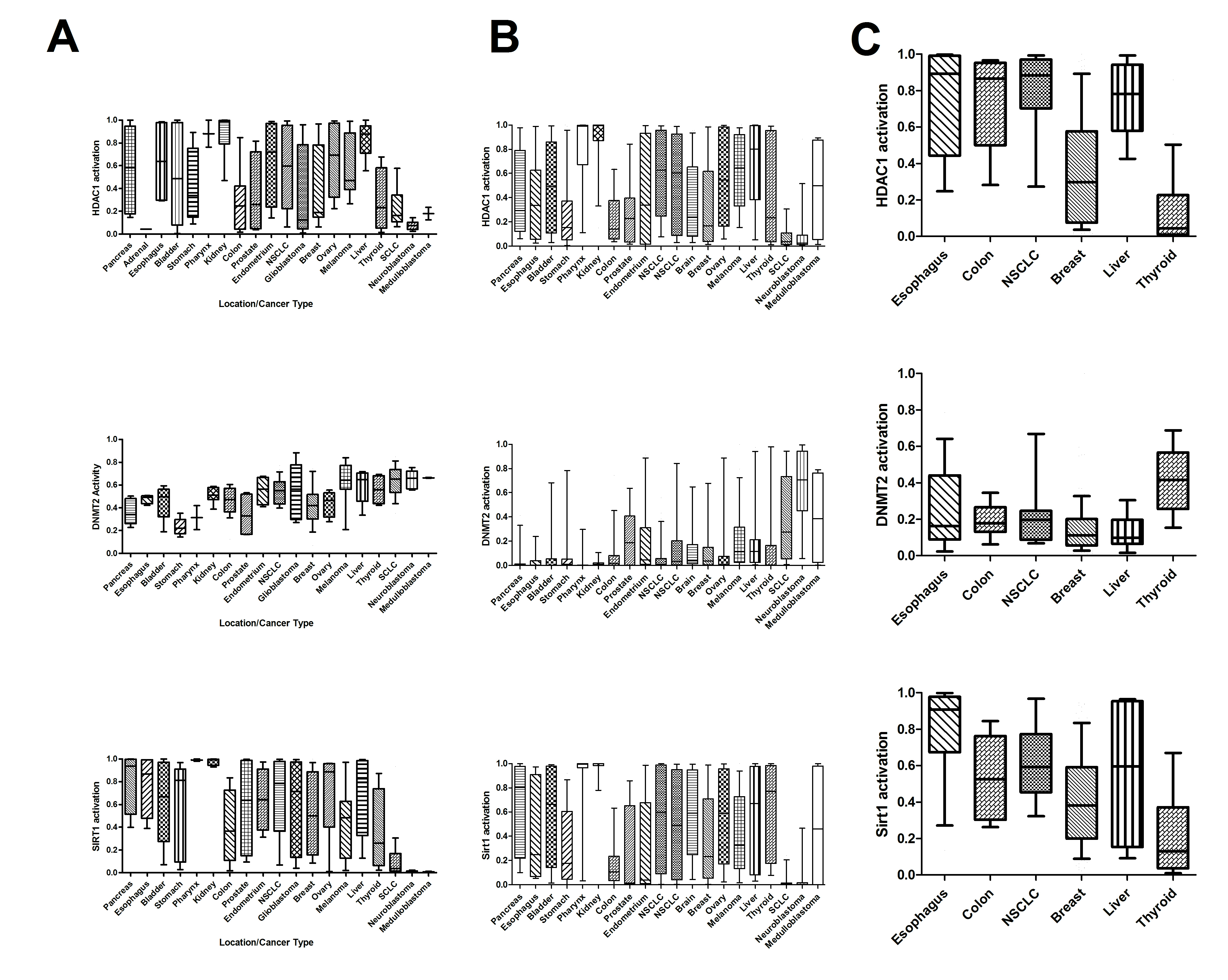

Supplement: Additional file 7: Figure S3 — Epigenetic pathway predictions for HDAC1, DNMT2, and SIRT1 in (A) GSK and (B) CCLE cell line collections. [file 1755-8794-6-35-S7.jpeg]
