# Supplementary material for: Genomic pathway analysis reveals that EZH2 and HDAC4 represent mutually exclusive epigenetic pathways across human cancers
Source: BMC Med Genomics. 2013 Sep 30;6:35. doi: 10.1186/1755-8794-6-35 (PMC3850967; doi:10.1186/1755-8794-6-35)
Supplement: Additional file 2: Figure S1 — Supplementary methods with detailed instructions for running pathway predictions. [file 1755-8794-6-35-S2.doc]

SUPPLEMENTAL METHODS

I. Code Narrative for “Genomic Pathway Analysis Reveals that EZH2 and HDAC4 Represent Mutually Exclusive Epigenetic Pathways Across Human Cancers”

The analyses were performed using MATLAB 7 with the Bioinformatics and Statistical toolboxes installed.

All analyses were performed on a UNIX server.

All gene expression data used in the study were arrayed on the Affymetrix HU133 platform.

1. Development and Validation of Pathway Signatures.

This section will detail the steps taken to develop and validate the pathway signatures utilized in the study. We will detail the methods used to prepare the data, and the process used to generate the initial signature.

A. Data Preparation:

The .CEL files for all five pathway signatures were MAS5 normalized using Affymetrix Expression Console (ver1.0) and the resulting output file was saved as pathways_mas5.tab.

A dictionary file was created for each signature. The files are named signature_dict_v2_*.tab, where * is the name of the signature:EZH2, HDAC4, HDAC1, SIRT1, or DNMT2. The first column lists the name of the signatures. The second column lists the names of the treated cell lines. The third column lists the names of the control cell lines.

A list of all the probes on the U133A chip was saved in a file called affxid.order. All probes on the chip were used for all of the signatures except for the EZH2 signature. The EZH2 signature uses a probe list file called affxid_noe.order, in which the probe for the EZH2 gene itself has been removed in order to prevent this probe from dominating the signature.

C. Generation of the Epigenetic Signature:

The perl and matlab library files needed to run the program are saved in perl_library.zip and matlab.zip, respectively. Parameter files were created with file locations and parameter settings called default*.config, where * is replaced by the name of the signature to be run. In UNIX, the binary regression wrapper was called using the command perl run_project.pl –c default.config. The wrapper first transformed both the signature and dataset CEL files by log based 2 and then quantile normalized them. A script to perform distance weighted discrimination was called in MATLAB. The output from this script was then filtered to remove probe sets with low expression or low variability and then fed into the binary regression program, binreg.

The signature probe list and regression weights are reported by BINREG in the output file entitled genecoefficient.txt and the image intensity display (or heatmap) of these values is reported in output figure 11.

The formal leave-one-out cross validation used to examine the stability and predictive capability of the model is reported by BINREG in the file entitled figure5.txt and the corresponding graph is reported in BINREG figure 5.

D Optimization of signature

Parameters were optimized in order to minimize the p-value of a t-test of the leave-one-out cross-validation predictions of the untreated and treated samples. The number of iterations was chosen so that predictions in a sample dataset were stable.

The parameters for the signatures are listed in the signature_parameters_master_file.xls.

E. Binary regression algorithm

The analysis of drug response made use of previously described statistical methods (Bild et al., 2006; Huang et al., 2003; West et al., 2001). In this analysis, a signature represents a group of genes that collectively exhibit a consistent pattern of expression and which enable distinction between two phenotypes. A metagene representing a group of genes that collectively demonstrate a consistent pattern of expression for a specific phenotype is identified from the training data (Phenotype versus Phenotype B). Each signature summarizes its constituent genes as a single expression profile and is derived from the first principal component of that gene set. This factor corresponds to the largest singular value as determined by singular value decomposition (SVD). Bayesian methods are then used to estimate binary probit regression models based on a given set of expression vectors (values across metagenes) derived from the training data. Application of these models to an independent validation dataset enables the evaluation of predictive probabilities of each of the two phenotypic states for each sample in the validation dataset. In these analyses, gene selection and identification is based solely on the training data and metagene values are computed using the principal components of the training data ensuring reproducibility of the signature irrespective of the composition of the validation dataset. Bayesian fitting of binary probit regression models to the training data enable assessment of the relevance of the metagene signature in within-sample classification as well as estimation and uncertainty assessment for the binary regression weights. This results in the mapping of metagenes to probabilities of relative pathway status.

Evaluation of independent tumor or cell line samples results in the prediction of relative pathway status generating estimated relative probabilities, and associated measures of uncertainty, of activation or deregulation for each sample in the validation dataset.

To ensure that over-fitting does not occur in the generation of each signature, a leave-one-out cross validation was performed for each set of training data to examine the stability and predictive capabilities of our model. In this analysis, each sample is left out, one at a time, of the dataset and the model was refitted (both the metagene factors and the partitions used) using the remaining samples. The phenotype of the held out sample is predicted and the certainty of the classification calculated.

When multiple datasets are combined, the probit regression output for each dataset is linearly transformed to a relative probability so that the maxmimum probability is 1 and the minimum probability is zero in order to minimize cross-dataset variability. The formula for the transformation for a value x_i in the probit output for dataset X is x_i -> (x_i – minimum(X))/(maximum(X) – minimum(X)).

References

Bild A, Yao G, Chang JT, Wang Q, Potti A, Chasse D et al (2006). Oncogenic pathway signatures in human cancers as a guide to targeted therapies. Nature 439: 353-357.

Dauer DJ, Ferraro B, Song L, Yu B, Mora L, Buettner R et al (2005). Stat3 regulates genes common to both wound healing and cancer. Oncogene 24: 3397-3408.

Frey BJ, Dueck D (2007). Clustering by passing messages between data points. Science 315: 972-976.

Ghosh D, Chinnaiyan AM (2002). Mixture modeling of gene expression data from microarray experiments. Bioinformatics 18: 275-286.

Golub TR, Slonim DK, Tamayo P, Huard C, Gaasenbeek M, Mesirov JP et al (1999). Molecular classification of cancer: class discovery and class prediction by gene expression monitoring. Science 286:

531-537.

Huang E, Ishida S, Pittman J, Dressman H, Bild A, Kloos M et al (2003). Gene expression phenotypic models that predict the activity of oncogenic pathways. Nat Genet. 34: 226-230.

Kim-Anh D, Muller P, Tang F (2005). A Bayesian mixture model for differential gene expression. J.

Royal Stat. Soc. 54: 627-654.

McLauchlan GJ, Bean RW, Peel D (2002). A mixture model based approach to the clustering of microarray expression data. Bioinformatics 18: 413-422.

Miscia S, Marchisio M, Grilli A, Di Valerio V, Centurione L, Sabatino G et al (2002). Tumor Necrosis Factor alpha (TNF-alpha) Sctivates Jak1/Stat3-Stat5B Signaling through TNFR-1 in Human B Cells. Cell Growth & Differentiation 13: 13-18.

Mori S, Rempel RE, Chang JT, Yao G, Lagoo AS, Potti A et al (2008). Utilization of pathway signatures

to reveal distinct types of B lymphoma in the Emu-myc model and human DLBCL. Cancer Res. 68:

8525-8534.

Potti A, Dressman HK, Bild A, Riedel RF, Chan G, Sayer R et al (2006). Genomic signatures to guide the

use of chemotherapeutics. Nat Med 12: 1294-1300.

Rouzier R, Perou CM, Symmans WF, Ibrahim N, Cristofanilli M, Anderson K et al (2005). Breast cancer

molecular subtypes respond differently to preoperative chemotherapy. Clin Cancer Res. 11: 5678-5685.

West M, Blanchette C, Dressman H, Huang E, Ishida S, Spang R et al (2001). Predicting the clinical

status of human breast cancer by using gene expression profiles. Proc Natl Acad Sci USA 98: 11462-

11467.
